# Prevalence and risk factors of nocturnal enuresis among children ages 5–12 years in Xi’an, China: a cross-sectional study

**DOI:** 10.1186/s12887-020-02202-w

**Published:** 2020-06-22

**Authors:** Hui-Mei Huang, Jing Wei, Shristi Sharma, Ying Bao, Fei Li, Jian-Wen Song, Hai-Bin Wu, Hong-Li Sun, Zhi-Juan Li, Huan-Nan Liu, Qian Wu, Hong-Li Jiang

**Affiliations:** 1 Department of Epidemiology and Biostatistics, School of Public Heath, Xi’an Jiaotong University, No.76 West Yanta Road, Xi’an, 710061 Shaanxi China; 2 Department of Renal Dialysis, The First Affiliated Hospital of Xi’an Jiaotong University, No.277 West Yanta Road, Xi’an, 710061 Shaanxi China; 3 Department of Nephrology, The Affiliated Children Hospital of Xi’an Jiaotong University, No.69 Xijuyuan Lane, Xi’an, 710002 Shaanxi China; 4 Department of Breast Cancer, Shaanxi Tumor Hospital, No.309 West Yanta Road, Xi’an, 710061 Shaanxi China; 5 Department of Dermatology, The Affiliated Children Hospital of Xi’an Jiaotong University, No.69 Xijuyuan Lane, Xi’an, 710002 Shaanxi China; 6 Department of Pediatric intensive care unit, The Affiliated Children Hospital of Xi’an Jiaotong University, No.69 Xijuyuan Lane, Xi’an, 710002 Shaanxi China; 7 Shaanxi Institute of Pediatric Diseases, The Affiliated Children Hospital of Xi’an Jiaotong University, No.69 Xijuyuan Lane, Xi’an, 710002 Shaanxi China

**Keywords:** Nocturnal enuresis, Children, Prevalence, Risk factors

## Abstract

**Background:**

Nocturnal enuresis (NE) has a negative impact on children’s health and imposes a long-term burden on families. With economic development and cultural improvements, parents and medical professionals pay more attention to NE. The aim of this study was to investigate the prevalence and risk factors of NE among children ages 5–12 years in Xi’an, China.

**Methods:**

A stratified cluster sampling method was used to conduct a cross-sectional study of NE in 10 kindergartens and 20 primary schools in Xi’an. We used univariate analysis to compare the prevalences of characteristics such as gender, duration of disposable diaper (DD) use, toilet training onset time, daily living habits, academic performance, and family history of NE. Logistic regression analysis was used to calculate odds ratio and to determine risk factors of NE.

**Results:**

The study included 6568 children ages 5–12 years, of which 262 (3.99%) had NE. The prevalence rates of NE decreased with age, with the highest prevalence at age 5 (9.09% for boys; 6.03% for girls). However, the prevalence increased with duration of DD use. Children experienced more NE if they never accepted toilet training (7.83%) or if they drank sugary beverages during the day (5.36%). Sleep disorders, sweets intake, drinking low amounts of plain water during the day, and family history of NE, were statistically associated with NE.

**Conclusion:**

NE was closely associated with a family history of NE, being male, long-term use of DD, delayed toilet training, drinking sugary beverages and/or consuming little plain water, and sleep disorders. A supportive parental attitude towards NE and timely medical treatment can improve the quality of life of enuretic children.

## Background

Childhood nocturnal enuresis (NE) refers to the symptoms of intermittent urinary incontinence during sleep, at a minimum age of 5 years, with a minimum duration of three months, and a minimum of one episode per month [1]. The pathogenesis of NE is complex and multifactorial. Contributing elements include family history of NE, changes in antidiuretic hormone secretion rhythm, sleep disorders, retarded bladder maturity, immature nerve development, psychology, and environment [2–4].

In Egypt, Hamed et al. found the prevalences of NE in rural and urban areas were 17.5 and 18.4% [2]. In Turkey, the overall occurrence of NE was 7.5–16.2% [3, 5, 6], and, in Iran, the prevalence was 8–18.7% [4, 7]. Furthermore, Ramírez-Backhaus et al. found that the prevalence of NE in Spanish school-age children was 7.8% [8]. In China, NE prevalence was found to vary by region, ranging from 4.07 to 10.3% [9, 10], and NE occurred more frequently with boys [3, 11].

The risk factors for NE include long-term use of disposable diaper (DD) [10], being male, difficulty in awakening at night [11], mental stress, poverty [12], and family environment [13]. With the use of DD, toilet training may be delayed. In western countries, parents initiate toilet training depending on a child’s physical and psychological development, usually between the age of 21 and 36 months [14]. Because of the advent of DD, more and more parents start toilet training after their children’s second birthday. Some children have never been trained. However, in traditional Chinese culture, parents usually begin toilet training before children’s first birthday. In rural areas, some children even receive toilet training within 6 months of birth. A study has confirmed that infants who use DD and receive toilet training within the age of 12 months have better control over urination than those who delay toilet training [15]. Although NE does not cause physical problems, and 10–15% of enuretic children recover spontaneously, parents and medical practitioners cannot ignore the negative influence of NE on a child’s social, emotional, and psychological development [16, 17]. Also, NE brings greater economic burden and mental pressure to families. However, most parents have a casual attitude towards this condition, and lack awareness of medical treatment, which ultimately causes delay in treatment [18]_._ Fortunately, economic development and cultural improvement are promoting increased attention to NE among parents and medical professionals. Economic development may make parents more attentive towards child’s health, and their awareness of NE makes them consider NE to be a developmental problem for their child. Moreover, cultural awareness has led to greater acceptance of NE as a problem, which allows many children and/or parents to open about the problem.

To date, there are no large-scale epidemiological surveys of NE in Xi’an. There is a small-scale survey conducted in 2005 that showed a 5.2% (84/1626) prevalence of NE among school-age children [19]. Thus, we sought to investigate the prevalence of NE in children ages 5–12 years in Xi’an and assess potential risk factors of NE, to provide baseline data for prevention and treatment.

## Methods

### Study participants

From December 2018 to January 2019, we conducted a cross-sectional survey of NE among children ages 5–12 years in five regions throughout Xi’an. Ten kindergartens and 20 primary schools were selected by stratified cluster sampling. NE was diagnosed according to ICCS-2016 [1].

### Survey method

The investigators in each school received standardized training. They obtained informed consent from a parent or guardian on behalf of all participants. Parents received questionnaires including informed consent at the parent-teacher conference, using the “Questionnaire Star” software. They completed the questionnaires in the class as a unit.

### Content of questionnaire

To design the questionnaire, we referred to research of Ma and Tang [19, 20]. The questionnaire included an introduction that explained the importance of the study.

The questionnaire had five parts: general items, life behavior characteristics, quality of life, family history of NE, and parental attitude towards NE. “The Questionnaire for the Assessment of Children’s General Quality of Life” was compiled by referring to similar studies from other countries, taking into account current conditions in China [19]. All answers were divided according to increasing frequency of occurrence. Referring to the WHOQOL-BREF scale [21], 25 items were ranked into four dimensions: physiology, psychology, social relations, and environment. All items had 1–5 points; items 3, 7, 9, 10, 11, 15, 18, 19, 21 and 23 were scored backwards. Children were divided into two age groups (5–6 and 7–12), when calculating quality of life scores. The higher the average score of each dimension, the better the quality of life.

### Statistical methods

The mean, standard deviation (SD), median, quartile range and frequency were used to describe the data. Two Independent Sample T test and the Mann-Whitney U test were used for analysis of quantitative data; otherwise, a Chi-squared test and Chi-squared test for trend were used to compare the distribution of categorical variables. Logistic regression analysis was used for multivariate analysis. A *P*-value < 0.05 was considered to be statistically significant. The Statistical Package for Social Sciences (SPSS; version 13.0, IBM, Armonk, NY, USA) was used for all statistical tests.

## Results

We distributed 6590 questionnaires; participants returned 6568 (99.67%) valid questionnaires, of which 3409 (51.90%) were from boys.

Table 1 lists the general characteristics of the study children. The average ages (±SD) of children with and without NE were 7.21 (±1.99) and 8.05 (±2.08) years (t = − 6.413, *P* < 0.001). Otherwise, between NE and non-NE groups, there were no significant differences in the distributions of mother’s age at delivery and parental education level.

**Table 1:** General characteristics of children ages 5–12 years

Factors		Total number	NE	non-NE	Prevalence(%)	*χ*^*2*^	*P*
Age		–	7.21 ± 1.99	8.05 ± 2.08	–	−6.413	< 0.001*
Gender	Male	3409	169	3240	4.96	17.356	< 0.001
	Female	3159	93	3066	2.94		
Educational level of father	Primary school	194	14	180	7.22	0.405	0.525**
	Junior high school	1761	65	1696	3.69		
	High school	1454	58	1396	3.99		
	Junior college	1538	62	1476	4.03		
	Bachelor degree	1348	55	1293	4.08		
	Postgraduate and above	273	8	265	2.93		
Educational level of mother	Primary school	238	16	222	6.72	0.085	0.770**
	Junior high school	1826	66	1760	3.61		
	High school	1463	58	1405	3.96		
	Junior college	1621	64	1557	3.95		
	Bachelor degree	1254	53	1201	4.23		
	Postgraduate and above	166	5	161	3.01		
Mother’s age at delivery	< 20 years old	63	5	58	7.94	0.732	0.392**
	20–29 years old	4957	186	4771	3.75		
	30–39 years old	1509	70	1439	4.64		
	≥40 years old	39	1	38	2.56		

Note: NE = nocturnal enuresis. Age is mean ± SD. * *P* value for Two Independent Sample T test, ** *P* value for Chi-squared test for trend, and other *P* value for Chi-squared test

**Fig. 1:**
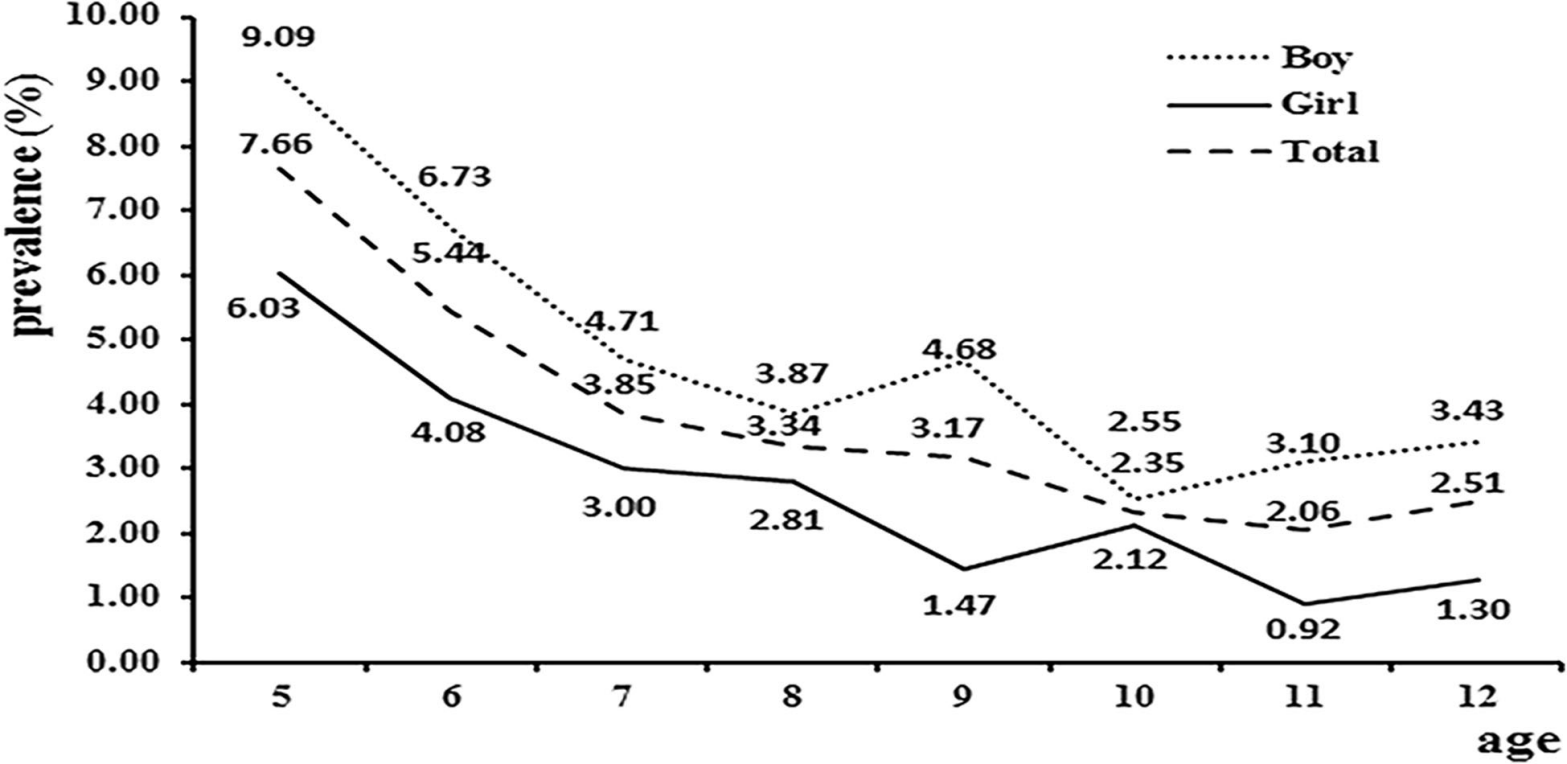
Prevalence of NE in children ages 5–12

The frequency of NE among children ages 5–12 was 3.99% (262/6568), with a higher prevalence among boys (4.96% vs 2.94%, *χ*^*2*^ = 17.356, *P* < 0.001,Table 1). The prevalence of NE in 5-year-old children was higher than the prevalence at older ages (9.09% for boys; 6.03% for girls). With increasing age, the prevalence rates of NE for boys and girls both showed downward trends (Fig. 1 and Supplementary Table 1, Additional File 1).

The prevalences of NE in five regions were as follows: urban area, 4.24%; eastern suburb, 3.00%; western suburb, 2.31%; southern suburb, 4.77%; northern suburb, 3.60%. The differences between regions were statistically significant (*χ*^*2*^ = 12.013, *P* = 0.017).

The prevalence of NE among children increased with duration of DD use. Statistically, the children who never accepted toilet training (7.83%) and those who drank sugary beverages during the day (5.36%) experienced more NE compared with others. Children who drank plain water and ate fruits before bed (4.42%), or had sleep disorders (5.98%), or who awakened with difficulty from sleep (4.43%) had more NE than children without these behaviors and symptoms. The prevalence of NE in children with poor academic performance and family history of NE was 13.49 and 18.04%, respectively (Table 2).

**Table 2:** Univariate analysis of associated factors of NE among children ages 5–12 years

Factors		Total number	NE	non-NE	Prevalence(%)	*χ*^*2*^	*P*
Delivery mode	Natural labor	3576	125	3451	3.50	4.992	0.025
	Caesarean birth	2992	137	2855	4.58		
Duration of using DD	Never use	994	19	975	1.91	131.360	< 0.001**
	0-1 year	2532	59	2473	2.33		
	> 1 but ≤2 years	2492	122	2370	4.90		
	> 2 but ≤3 years	454	32	422	7.05		
	> 3 but ≤4 years	67	16	51	23.88		
	> 4 years	29	14	15	48.28		
Toilet training onset time	< 6 months of age	2368	71	2297	3.00	20.940	< 0.001**
	6-12 months of age	2555	107	2448	4.19		
	13-18 months of age	998	37	961	3.71		
	> 18 months of age	149	8	141	5.37		
	never training	498	39	459	7.83		
Living habits during the day	Drinking sugary beverages	971	52	919	5.36	16.304	0.001
	Eating sweets	1645	74	1571	4.50		
	drinking low amounts of plain water(< 500 ml)	1505	68	1437	4.52		
	None of the above	2447	68	2379	2.78		
Drinking plain water and eating fruits before bed	No	2429	79	2350	3.25	5.462	0.019
	Yes	4139	183	3956	4.42		
The time to fall asleep	Before 21:00	1114	24	1090	2.15	14.757	< 0.001**
	21:00–23:00	5354	230	5124	4.30		
	After 23:00	100	8	92	8.00		
Awakening from sleep	Easy	2122	65	2057	3.06	7.017	0.008
	Difficult	4446	197	4249	4.43		
Sleep disorders	Yes	1773	106	1667	5.98	25.100	< 0.001
	No	4795	156	4639	3.25		
Playing games before bed	No	4384	161	4223	3.67	3.884	0.049**
	Sometimes	2103	96	2007	4.56		
	Often	81	5	76	6.17		
Academic performance	Good	4289	149	4140	3.47	17.053	< 0.001**
	General	2153	96	2057	4.46		
	Poor	126	17	109	13.49		
Family history of NE	No	4939	77	4862	1.56	390.83	< 0.001
	Not sure	1130	95	1035	8.41		
	Yes	499	90	409	18.04		

Note: NE = nocturnal enuresis; DD = disposable diaper. ** *P* value for Chi-squared test for trend, and other P value for Chi-squared test

Logistic regression analysis showed that being female, older and exhibiting good academic performance appeared to be protective factors for NE. Long-term use of DD (more than one year), delayed toilet training, drinking sugary beverages, consuming low amounts of plain water (< 500 ml) during the day, falling asleep late, sleep disorders, and family history of NE correlated positively with occurrence of NE (Table 3).

**Table 3:** Logistic regression analysis of associated factors of NE in children ages 5–12 years

Factors		B	SE	Wals	*P*	OR	95% *CI*
Gender	Male					1(ref)	
	Female	−0.444	0.145	9.416	0.002	0.641	0.483–0.852
Age	–	−0.173	0.037	21.492	< 0.001	0.841	0.782–0.905
Delivery mode	Natural labor					1(ref)	
	Caesarean birth	0.222	0.137	2.629	0.105	1.248	0.955–1.632
Duration of using DD	Never use					1(ref)	
	0-1 year	0.121	0.278	0.188	0.665	1.128	0.654–1.947
	> 1 but ≤2 years	0.734	0.267	7.575	0.006	2.084	1.235–3.516
	> 2 but ≤3 years	0.856	0.319	7.208	0.007	2.354	1.260–4.399
	> 3 but ≤4 years	2.055	0.419	24.080	< 0.001	7.806	3.435–17.737
	> 4 years	3.119	0.503	38.471	< 0.001	22.624	8.444–60.620
Toilet training onset time	< 6 months of age					1(ref)	
	6-12 months of age	0.257	0.167	2.369	0.124	1.293	0.932–1.795
	13-18 months of age	0.011	0.222	0.003	0.959	1.011	0.655–1.562
	> 18 months of age	−0.048	0.418	0.013	0.909	0.953	0.420–2.164
	Never training	0.557	0.240	5.376	0.020	1.745	1.090–2.793
Drinking plain water and eating fruits before bed	No					1(ref)	
	Yes	0.110	0.150	0.536	0.464	1.116	0.832–1.496
Living habits during the day	None of the following					1(ref)	
	drinking sugary beverages	0.604	0.212	8.112	0.004	1.830	1.207–2.773
	Eating sweets	0.308	0.187	2.705	0.100	1.361	0.943–1.965
	drinking low amounts of plain water(< 500 ml)	0.526	0.191	7.536	0.006	1.692	1.162–2.462
The time to fall asleep	Before 21:00					1(ref)	
	21:00–23:00	0.457	0.232	3.886	0.049	1.579	1.003–2.486
	After 23:00	0.604	0.479	1.595	0.207	1.830	0.716–4.677
Awakening from sleep	Easy					1(ref)	
	Difficult	−0.283	0.156	3.278	0.070	0.753	0.555–1.024
Sleep disorders	No					1(ref)	
	Yes	0.291	0.141	4.235	0.040	1.338	1.014–1.764
Playing games before bed	No					1(ref)	
	Sometimes	−0.068	0.147	0.212	0.645	0.934	0.700–1.247
	Often	−0.273	0.534	0.261	0.609	0.761	0.267–2.167
Academic performance	Poor					1(ref)	
	General	−1.033	0.328	9.937	0.002	0.356	0.187–0.677
	Good	−1.276	0.326	15.346	< 0.001	0.279	0.148–0.529
Family history of NE	No					1(ref)	
	Not sure	1.549	0.164	89.245	< 0.001	4.705	3.412–6.488
	Yes	2.458	0.172	204.34	< 0.001	11.680	8.336–16.355

Note: ref. = reference; NE = nocturnal enuresis; *CI* = confidence interval

Compared with children without NE, the quality of life scores of enuretic children in physiology and social relations were lower in two age groups (*P* < 0.05). As for the psychological field, there was a significant difference in quality of life scores between NE and non-NE groups, only in children ages 7–12 (36 vs 38, *P* < 0.001; Supplementary Table 2, Additional File 1).

## Discussion

Nocturnal enuresis (NE) is a common childhood condition. Its pathogenesis is complex and associated with many factors [20]. In 2005, the prevalence of NE among all primary school students in China was 4.6%. For selected cities, the prevalence of NE was 7.4% in Wuhan, 3.3% in Shanghai, and 5.2% in Xi’an [19]. However, compared to the findings of the 2005 study in Xi’an, our sample’s prevalence of NE was lower, 3.99%. This lower prevalence might have been due to improvements in income, parental occupation, parental education level, and living habits [9].

We found that the prevalence of NE was higher in boys than girls. The highest frequency was 9.09% for boys and 6.03% for girls at age 5. The incidence of NE is known to decrease gradually with age, and our results also showed the same downward trend. We also found that age was a protective factor for NE, consistent with previous research [2, 22]. Age may relate to decreasing rates of NE because a child’s central nervous system develops with increasing age, and the neural pathways that control urination can better regulate urination activities, manage the storage and discharge of urine at night, and reduce the occurrence of bed-wetting [23].

We also found that being female and having good academic performance were protective factors of NE, findings that agreed with another study [11]. Genetic factor plays a decisive role in NE [3], and the poor control of nervous system over the bladder at night may be more heritable in boys than in girls [24], so boys are more likely to wet their beds than girls. In addition, bed-wetting has a negative effect on the body and mind [16] and it results in poor sleep quality in children with NE; this effect may cause enuretic children to be self-conscious and unable to concentrate on their studies, leading to poor academic performance.

NE has been closely associated with DD use, family income, and parental education level [12, 25, 26]. However, in our study, the difference in parental education levels was not statistically significant. In addition, we found that the prevalence of NE varied with regions, with the highest in the southern suburb followed by the urban area. This regional variation might exist because of rapid development of the economy in the southern suburb and urban area. We also found that with the widespread use of DD, younger children had used it much longer than the older (Supplementary Table 3, Additional File 1). Due to increases in income, parents may prolong the use of DD and delay toilet training, thus promoting the occurrence of NE [9]. Joinson et al. [27] suggested that delayed toilet training (after 24 months) was likely to induce persistent urine accidents during the daytime. But Hodges et al. found that children with early (before 24 months) or late (after 36 months) toilet training had more complaints of daytime wetness than children with normal training [28]. The difference in the age of toilet training may be due to individual differences of children and different child-rearing concepts of parents in different cultures. Children who use DD at night may attain nighttime dryness later compared to those who do not use DD at night. Therefore, parents should be encouraged to watch for signs that their child wakes up in the night for urination or is staying dry during the night. These cues might indicate readiness for a trial without DD.

The occurrence of NE is also closely related to sleep disorders [29, 30]. If they play games before bed, children with poor sleep quality could aggravate their fatigue and easily have difficulty waking from sleep [26, 31], in turn, inducing bed-wetting.

In addition to heredity, daily living habits are associated with NE. We found that the occurrence of NE in children with a family history of NE was significantly higher than that in the general population. In addition, behavioral factors can also induce NE. If children drink much sugary beverages during the day, it may have diuretic effects and lead to bed-wetting at night.

Parents may not consider NE to be a problem that could be helped by a physician, thus, they ignore it. Of the 262 enuretic children in our study, only 78 sought medical care, and parents of 136 enuretic children blamed and scolded their children for bed-wetting (Supplementary Table 4, Additional File 1). Parents who do not seek medical treatment for their children usually resort to potentially harmful, non-medical measures to prevent bed-wetting, such as waking their children to urinate at night and limiting water and fruit intake before bed [32]. A study has confirmed that NE was closely associated with parental abuse and neglect, which severely damages children’s psychological development and family life quality [33].

NE has a negative impact on children and families [17]. We found that children with NE scored lower in quality of life than children without NE in physiological health and social relations. This phenomenon may have occurred because enuretic children slept on damp beds and were liable to become unwell because of poor physical resistance, in turn promoting a poor quality of life [19]. We also found that NE appeared to have a great impact on psychology among older children (7–12 years old). Children with NE may be derided and disliked by classmates and have low self-esteem, which may affect their ability to communicate and hinder their progress in toilet training and their psychological development [34]. NE imposes a long-term burden on families, which may cause parents to have negative feelings about their child.

The limitations are that the results were possibly compromised by the potential retrospective recall biases, such as memory bias of the parents’ report on the duration of using DD and age of toilet training onset. This study was performed in order to establish baseline data that would lead to more comprehensive studies on NE. The causes for NE may be multifactorial, and the medical community needs more prospective studies to verify its risk factors.

## Conclusion

This study was the first large-scale cross-sectional survey of NE in children, ages 5–12, in Xi’an, China. The major strengths of the study are the large sample size and its epidemiological and representative design because the sample covered five regions throughout Xi’an, including urban and rural areas. The prevalence of NE was 3.99%, lower than the frequency in 2005. Prevalence decreased with age and was higher in boys and in economically developed areas. NE was closely associated with a family history of NE, being male, long-term use of DD (more than one year), delayed toilet training, sugary beverage consumption, lack of plain water intake, and sleep disorders. Early toilet training, a helpful parental attitude towards NE, and concern for children’s physical and mental health are likely to improve the quality of life for children who experience NE.

## Supplementary information

**Additional file 1.** Supplementary Table 1 Prevalence of NE in boys and girls ages 5–12. Supplementary Table 2 Quality of life scores of children in two age groups. Supplementary Table 3 Age of children in DD use groups. Supplementary Table 4 Parental attitude and behaviors towards enuretic children. Description of data: The four supplementary tables in Additional File 1 contain additional information that supports our findings in the main manuscript.

## Data Availability

The datasets used and/or analyzed during the current study available from the corresponding author on reasonable request.

## Abbreviations

NE: Nocturnal enuresis
DD: Disposable diaper
ref.: Reference
SD: Standard deviation
CI: Confidence interval
