# Supplementary material for: Prevalence and risk factors of nocturnal enuresis among children ages 5–12 years in Xi’an, China: a cross-sectional study
Source: BMC Pediatr. 2020 Jun 22;20:305. doi: 10.1186/s12887-020-02202-w (PMC7310244; doi:10.1186/s12887-020-02202-w)
Supplement: Supplementary file 1 — Additional file 1. Supplementary Table 1 Prevalence of NE in boys and girls ages 5–12. Supplementary Table 2 Quality of life scores of children in two age groups. Supplementary Table 3 Age of children in DD use groups. Supplementary Table 4 Parental attitude and behaviors towards enuretic children. Description of data: The four supplementary tables in Additional File 1 contain additional information that supports our findings in the main manuscript. [file 12887_2020_2202_MOESM1_ESM.docx]

**Supplementary tables:**

**Supplementary Table 1** Prevalence of NE in boys and girls ages 5-12

| age | Boy | | |  | Girl | | |  | Total | | |
| --- | --- | --- | --- | --- | --- | --- | --- | --- | --- | --- | --- |
|  | NE | children(N) | prevalence(%) |  | NE | children(N) | prevalence(%) |  | NE | children(N) | prevalence(%) |
| 5 | 38 | 418 | 9.09 |  | 22 | 365 | 6.03 |  | 60 | 783 | 7.66 |
| 6 | 40 | 594 | 6.73 |  | 23 | 564 | 4.08 |  | 63 | 1158 | 5.44 |
| 7 | 25 | 531 | 4.71 |  | 16 | 534 | 3.00 |  | 41 | 1065 | 3.85 |
| 8 | 19 | 491 | 3.87 |  | 14 | 498 | 2.81 |  | 33 | 989 | 3.34 |
| 9 | 18 | 385 | 4.68 |  | 5 | 341 | 1.47 |  | 23 | 726 | 3.17 |
| 10 | 11 | 431 | 2.55 |  | 8 | 377 | 2.12 |  | 19 | 808 | 2.35 |
| 11 | 11 | 355 | 3.10 |  | 3 | 326 | 0.92 |  | 14 | 681 | 2.06 |
| 12 | 7 | 204 | 3.43 |  | 2 | 154 | 1.30 |  | 9 | 358 | 2.51 |

**Supplementary Table 2** Quality of life scores of children in two age groups

|  | Dimension | NE | | non-NE | | *Z* | *P**** |
| --- | --- | --- | --- | --- | --- | --- | --- |
| Age group |  | Median | Quartile range(Q_1_,Q_3_) | Median | Quartile range(Q_1_,Q_3_) |  |  |
| 5-6 years old | Physiological field | 10 | 2(9,11) | 11 | 2(10,12) | -3.367 | 0.001 |
|  | Environmental field | 4 | 2(3,5) | 4 | 2(3,5) | -1.204 | 0.229 |
|  | Social relations | 41 | 7(38,45) | 43 | 6(40,46) | -2.891 | 0.004 |
|  | Psychological field | 40 | 6(37,43) | 40 | 5(38,43) | -0.392 | 0.695 |
|  |  |  |  |  |  |  |  |
| 7-12 years old | Physiological field | 10 | 2(9,11) | 11 | 2(10,12) | -4.029 | <0.001 |
|  | Environmental field | 4 | 2(3,5) | 4 | 2(3,5) | -0.726 | 0.468 |
|  | Social relations | 40 | 7(36,43) | 42 | 6(39,45) | -5.566 | <0.001 |
|  | Psychological field | 36 | 6(34,40) | 38 | 7(35,42) | -3.683 | <0.001 |

Note: NE = nocturnal enuresis; *** *P* value for Mann-Whitney U test.

**Supplementary Table 3** Age of children in DD use groups

| Duration of using DD | N | Age (year) |
| --- | --- | --- |
| Never use | 994 | 9.06 ± 1.98 |
| 0-1year | 2532 | 8.18 ± 2.03 |
| >1 but ≤ 2years | 2492 | 7.64 ± 2.00 |
| >2 but ≤ 3years | 454 | 7.11 ± 2.04 |
| >3 but ≤ 4years | 67 | 6.85 ± 1.99 |
| >4years | 29 | 6.14 ± 1.43 |

Note: age is mean ± SD.

**Supplementary Table 4** Parental attitude and behaviors towards enuretic children

|  | Seek medical treatment | |  | Scold and punish children | |
| --- | --- | --- | --- | --- | --- |
|  | N | % |  | N | % |
| No | 184 | 70.23 |  | 126 | 48.09 |
| Yes | 78 | 29.77 |  | 136 | 51.91 |
